# SMAD6 transduces endothelial cell flow responses required for blood vessel homeostasis

**DOI:** 10.1007/s10456-021-09777-7

**Published:** 2021-03-29

**Authors:** Dana L. Ruter, Ziqing Liu, Kimlynn M. Ngo, Shaka X, Allison Marvin, Danielle B. Buglak, Elise J. Kidder, Victoria L. Bautch

**Affiliations:** 1 Department of Biology, University of North Carolina at Chapel Hill, Chapel Hill, USA; 2 Lineberger Comprehensive Cancer Center, Chapel Hill, USA; 3 Department of Chemistry, University of North Carolina, Chapel Hill, USA; 4 Curriculum in Cell Biology and Physiology, Chapel Hill, USA; 5 McAllister Heart Institute, University of North Carolina, Chapel Hill, USA; 6 Department of Biology, CB#3280, University of North Carolina at Chapel Hill, Chapel Hill, NC 27599 USA

**Keywords:** Endothelial cells, Blood vessels, Blood flow, Notch, SMAD, BMP, PCDH12

## Abstract

Fluid shear stress provided by blood flow instigates a transition from active blood vessel network expansion during development, to vascular homeostasis and quiescence that is important for mature blood vessel function. Here we show that SMAD6 is required for endothelial cell flow-mediated responses leading to maintenance of vascular homeostasis. Concomitant manipulation of the mechanosensor Notch1 pathway and SMAD6 expression levels revealed that SMAD6 functions downstream of ligand-induced Notch signaling and transcription regulation. Mechanistically, full-length SMAD6 protein was needed to rescue Notch loss-induced flow misalignment. Endothelial cells depleted for SMAD6 had defective barrier function accompanied by upregulation of proliferation-associated genes and down regulation of junction-associated genes. The vascular protocadherin PCDH12 was upregulated by SMAD6 and required for proper flow-mediated endothelial cell alignment, placing it downstream of SMAD6. Thus, SMAD6 is a required transducer of flow-mediated signaling inputs downstream of Notch1 and upstream of PCDH12, as vessels transition from an angiogenic phenotype to maintenance of a homeostatic phenotype.

**Supplementary Information:**

The online version contains supplementary material available at 10.1007/s10456-021-09777-7.

## Introduction

Blood vessel formation intersects with tissue metabolic needs and architecture in multiple ways, including remodeling of the initial vascular plexus via shear stress provided by blood flow [1–3]. Endothelial cells orchestrate this remodeling and eventual transition to vascular homeostasis, as elevated heart pumping and blood volume increase fluid shear stress [4–7]. In contrast to the sprouting and blood vessel remodeling that characterize the “activated” endothelium of development, endothelial cells become quiescent under laminar flow at steady state. This transition is an important prerequisite for endothelial cell functions involved in homeostasis, including formation of a vascular barrier that regulates oxygen and nutrient exchange [8–11]. As homeostasis is induced, endothelial cells become less proliferative and remodel their cytoskeleton and junctions to align parallel to the flow vector, presumably to reduce flow-induced cellular stress [12–16]. The importance of this response is highlighted by the association of arterial laminar flow with protection from atherosclerosis, and the correlation between disturbed flow and atheroprone regions of vessels [17, 18]. Although several cell-surface protein complexes function as direct endothelial cell mechanosensors [19], and even more signaling pathways have flow-responsive components, how these sensors and pathways transduce flow-mediated inputs to set up and maintain vessel homeostasis remains poorly understood.

Notch and BMP are two pathways involved in endothelial cell flow responses. Notch1 was recently identified as a direct flow mechanosensor, and endothelial deletion of Notch1 leads to flow misalignment, increased permeability, and atherosclerotic plaque formation [20–22]. More generally, Notch signaling is upregulated in arteries, which are exposed to higher shear stress than veins of equivalent diameter, and Notch is thought to be responsible for maintaining arterial differentiation [23–25]. Although BMP pathway components are not direct mechanosensors, some are flow-regulated [4]; BMP signaling regulates blood vessel formation and function in complex and context-dependent ways [26–29], with proangiogenic signaling downstream of ligands such as BMP2 and BMP6 countered by antiangiogenic signaling downstream of BMP9 and/or BMP10. Human mutations in the Type 1 receptor Alk1/ACVRL1, the co-receptor Endoglin, or a common signaling component SMAD4 lead to Hereditary Hemmorrhagic Telangiectasia (HHT) that is characterized by arterio-venous malformations (AVMs) and hemorrhage [30–32]. AVM formation requires genetic loss of BMP signaling, blood flow, and a third proangiogenic or pro-inflammatory stimulus [33–36], suggesting an intimate relationship between BMP signaling and flow responses in blood vessels.

There are several intersection points between Notch and BMP signaling in endothelial cells. For example, transcriptional responses are altered in complex and interdependent ways when both Notch and BMP signaling are activated, leading to cooperative effects on signaling and endothelial cell quiescence [37–39]. We showed that proangiogenic endothelial cell BMP signaling in vitro and in vivo is regulated in part by an intracellular negative regulator of BMP signaling, SMAD6 [40]. SMAD6 expression is upregulated by laminar blood flow and genetic loss leads to vascular hemorrhage [41, 42], but whether it functions in the transition to stable homeostasis is not known. Here, we reveal a requirement for SMAD6 in the flow-mediated alignment of endothelial cells, homeostatic quiescence, and barrier function downstream of the mechanosensor Notch1. Flow-induced endothelial cell-cell junction gene expression required SMAD6, and a SMAD6-regulated protocadherin affected homeostatic flow responses. Thus, SMAD6 is a required transducer of endothelial flow-mediated responses instigated by Notch signaling and required for maintenance of vascular homeostasis.

## Results

### SMAD6 is required for homeostatic laminar flow-induced endothelial cell alignment and polarization

To begin to understand how endothelial cell flow-mediated responses that characterize vascular homeostasis are maintained, we asked whether an inhibitory SMAD, SMAD6, regulates endothelial cell responses to extended periods of laminar shear stress. *Smad6*^*−/−*^ mutant embryos and pups with a knock-in *lacZ* reporter in the *Smad6* locus strongly express *lacZ* in endothelial cells of embryonic and early post-natal arteries, but not veins of similar diameter and stage, after the onset of blood flow [42, 43]. Since shear stress induced by laminar flow is higher in arteries, this suggests that endothelial SMAD6 expression is induced by homeostatic laminar flow. Thus, we chose conditions of 15 dynes/cm^2^ (d/cm^2^) for 72 h for analysis of SMAD6 function in homeostatic arterial flow responses. Under these conditions, both human umbilical vein endothelial cells (HUVEC) and human arterial endothelial cells (HAEC) showed a two to threefold increase in Smad6 RNA (Supp. Fig 1A, B), consistent with a previous report [41].

**Fig. 1:**
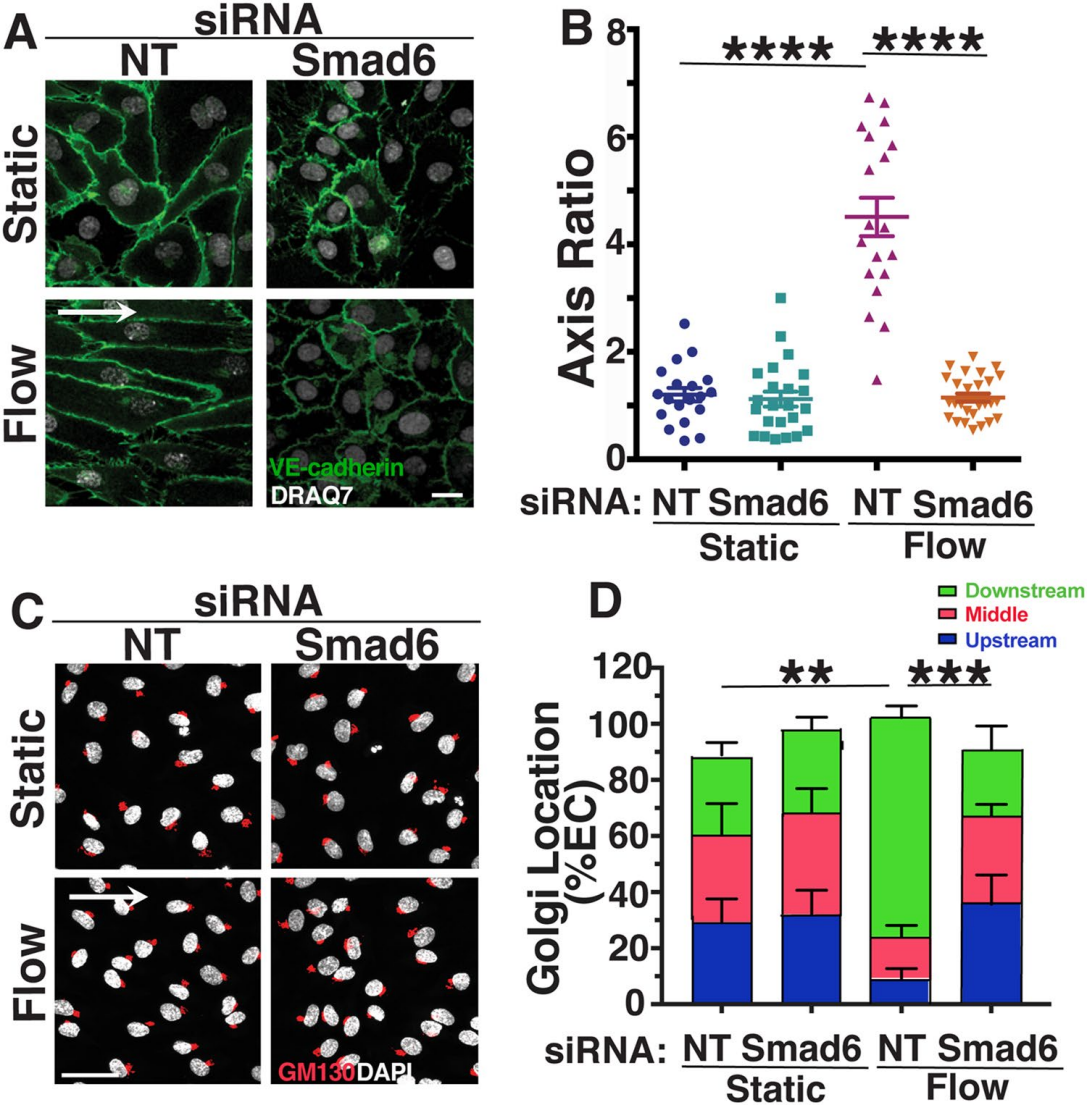
SMAD6 is required for homeostatic endothelial cell flow-mediated alignment and polarization. **a** Representative panels of HUVEC stained with VE-cadherin (green, junctions) and DRAQ7 (white, nucleus) under indicated conditions with indicated treatments. White arrow, flow vector. Scale bar, 20 μM. **b** Quantification of cell axis ratio. Statistical analysis, One-way ANOVA; ****p ≤ 0.0001. *N* = 3, representative experiment shown. **c** Representative panels of HUVEC stained with GM130 (red, Golgi) and DAPI (white, nucleus) under indicated conditions with indicated treatments. White arrow, flow vector. Scale bar, 50 μM. **d** Quantification of Golgi localization relative to nucleus in indicated conditions. ≥ 30 cells/condition were analyzed. Statistical analysis, One-way ANOVA; **p ≤ 0.01; ***p ≤ 0.001. *NT* non-targeting (control) siRNA

We next examined the function of SMAD6 in endothelial cell responses to homeostatic laminar flow, by interrogating HUVEC and HAEC depleted for Smad6 RNA via knockdown (KD) and exposed to flow. We found that both venous and arterial endothelial cells with reduced Smad6 RNA levels failed to align under homeostatic laminar flow, as indicated by cell axis ratio and nuclear displacement angle measured parallel to the direction of flow (Fig. 1a, b, Supp. Fig. 1C–F). Endothelial cell misalignment in response to homeostatic laminar flow occurred with multiple siRNAs targeting Smad6 (Smad6-1 and Smad6-2), including a Smad6 siRNA pool (Smad6-3) (Supp. Fig. 1G, H). Both venous and arterial endothelial cells depleted for Smad6 also had mis-positioned Golgi and centrosomes under flow compared to controls (Fig. 1c, d, Supp. Fig. I–K, not shown), indicating that SMAD6 is important for endothelial cell polarization in response to homeostatic laminar flow. These results show that endothelial cell SMAD6 expression is flow-regulated and induced by homeostatic laminar flow, and that SMAD6 is functionally required for homeostatic flow-mediated endothelial cell alignment and polarization.

### SMAD6 is required downstream of Notch for flow-mediated alignment of endothelial cells

**Fig. 2:**
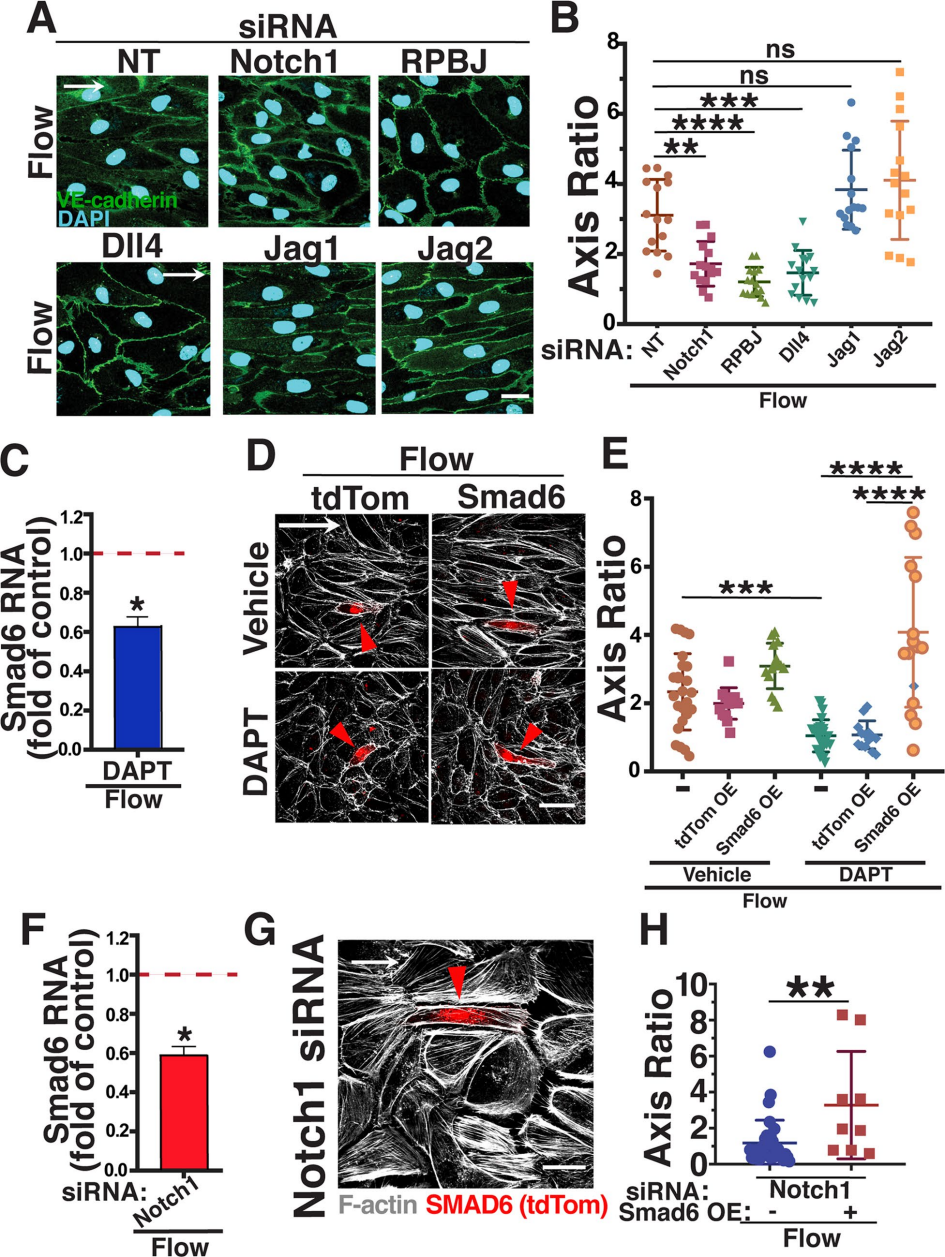
SMAD6 is downstream of Notch in homeostatic endothelial cell flow-mediated alignment. **a** Representative panels of HUVEC stained with VE-cadherin (green, junctions) and DAPI (blue, nucleus) under indicated conditions with indicated treatments. White arrow, flow vector. Scale bar, 20 μM. **b** Quantification of cell axis ratio. Statistical analysis, One-way ANOVA; **p ≤ 0.01; ***p ≤ 0.001; ****p ≤ 0.0001. *N* = 3, representative experiment shown. **c** qPCR RNA levels (normalized to vehicle control under flow) of SMAD6 in HUVEC treated with DAPT under flow. Statistical analysis, Student’s *t*-test; *p ≤ 0.05. **d** Representative panels of HUVEC stained with Phalloidin (F-actin, white) with indicated treatments and expression constructs (Empty Vector (EV) or SMAD6) under flow conditions. White arrow, flow vector. Red arrowhead, positive HUVEC. Scale bar, 50 μM. **e** Quantification of cell axis ratio. Statistical analysis, One-way ANOVA; ***p ≤ 0.001; ****p ≤ 0.0001. *N* = 3, representative experiment shown. **f** qPCR RNA levels (normalized to vehicle control under flow) in HUVEC treated with Notch1 siRNA under flow. Statistical analysis, Student’s *t*-test; *p ≤ 0.05. **g** Representative panels of HUVEC stained with Phalloidin (F-actin, white) with indicated treatments and over-expression construct (SMAD6) under flow conditions. White arrow, flow vector. Red arrowhead, positive EC. Scale bar, 20 μM. **h** Quantification of cell axis ratio. Statistical analysis, One-way ANOVA; **p ≤ 0.01. *N* = 3, representative experiment shown

SMAD6 is both a negative regulator of BMP signaling and a transcriptional target of the pathway [38, 44–46]. Consistent with the importance of canonical BMP signaling for endothelial cell flow alignment [47], endothelial cells were misaligned under homeostatic flow when incubated with the BMP inhibitor Crossveinless-2 (CV2) (Supp. Fig. 2A, B). However, since BMP receptors are not identified as direct mechanotransducers of flow-mediated signals, we searched for another pathway more directly linked to mechanotransduction that regulates SMAD6. Notch1 is a direct mechanotransducer of flow-mediated signaling [21, 22], and Notch regulates SMAD6 expression under static (non-flow) conditions [40], leading us to hypothesize that SMAD6 functions downstream of Notch in endothelial cell responses to homeostatic laminar flow. Reduced Notch signaling, via siRNA depletion of Notch1 or by treatment with the γ-secretase inhibitor DAPT which blocks Notch signaling, prevented endothelial cell alignment under homeostatic laminar flow (Fig. 2a, b, Supp. Fig. 2C, D). The misalignment induced by reduced Notch was accompanied by reduced expression of Smad6 RNA relative to controls under flow (Fig. 2c, f), indicating that SMAD6 expression levels are regulated downstream of Notch in endothelial cells and are important for homeostatic flow alignment. Consistent with this idea, ectopic SMAD6 expression rescued the flow-mediated misalignment of endothelial cells downstream of reduced Notch. Upon Notch blockade via DAPT, control endothelial cells transfected with empty vector remained misaligned when exposed to homeostatic laminar flow and did not differ from nearby untransfected cells; in contrast, similarly treated endothelial cells expressing SMAD6 aligned in response to homeostatic laminar flow while nearby untransfected cells remained misaligned (Fig. 2d, e). Endothelial cells over-expressing SMAD6 after Notch1 KD also aligned to homeostatic laminar flow while nearby cells remained misaligned (Fig. 2g, h). Thus SMAD6 over-expression rescued Notch loss-induced endothelial cell misalignment, indicating that SMAD6 is a functional effector of Notch-mediated homeostatic flow alignment in endothelial cells.

We next asked which Notch signaling components are required for homeostatic endothelial cell flow alignment. RPBJ is a transcriptional co-activator required for canonical downstream Notch signaling, and endothelial cells with reduced levels of RPBJ had reduced expression of Smad6 RNA (Supp. Fig. 2E). Moreover, RPBJ knockdown led to misalignment of both arterial and venous endothelial cells in response to homeostatic laminar flow (Fig. 2a, b; Supp. Figure 2F–I). Several Notch ligands are expressed in endothelial cells and implicated in Notch responses to flow. Reduced levels of Dll4, but not Jagged1 or Jagged2, resulted in endothelial cells misaligned in response to homeostatic laminar flow (Fig. 2a, b; Supp. Fig. 2F, G), indicating that Dll4-mediated activation of Notch1 signaling is important for homeostatic endothelial cell flow alignment via canonical Notch signaling.

**Fig. 3:**
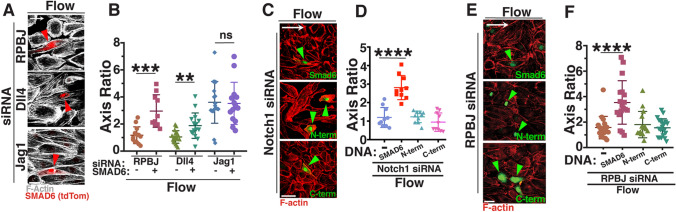
Full-length SMAD6 rescues Notch loss-induced endothelial cell homeostatic flow mis-alignment. **a** Representative panels of HUVEC stained with Phalloidin (F-actin, white) with indicated siRNA treatments and SMAD6 expression construct under flow conditions. White arrow, flow vector. Red arrowheads, positive EC. Scale bar, 50 μM. **b** Quantification of cell axis ratio. Statistical analysis, One-way ANOVA; **p ≤ 0.01; ***p ≤ 0.001; *ns* not significant. *N* = 3, representative experiment shown. **c** Representative panels of HUVEC stained with Phalloidin (F-actin, red) with Notch1 siRNA treatments and expression constructs (full-length SMAD6, N-terminal SMAD6, or C-terminal SMAD6; green) under flow conditions. White arrow, flow vector. Green arrowhead, positive EC. Scale bar, 50 μM. **d** Quantification of cell axis ratio. Statistical analysis, One-way ANOVA; ****p ≤ 0.0001. *N* = 3, representative experiment shown. **e** Representative panels of HUVEC stained with Phalloidin (F-actin, red) with RPBJ siRNA treatment and expression constructs (full-length SMAD6, N-terminal SMAD6, or C-terminal SMAD6; green) under flow conditions. White arrow, flow vector. Green arrowhead, positive EC. Scale bar, 50 μM. **f** Quantification of cell axis ratio. Statistical analysis, One-way ANOVA; ****p ≤ 0.0001. *N* = 3, representative experiment shown

The misalignment induced by RPBJ or Dll4 knockdown was rescued by expression of SMAD6 in HUVEC (Fig. 3a, b), and SMAD6 expression also rescued alignment of arterial endothelial cells with homeostatic laminar flow after RPBJ KD (Supp. Fig. 2H, I), suggesting that Notch regulation of SMAD6 expression is an important component of endothelial cell responses to homeostatic flow. The SMAD6 protein consists of two major domains connected by a linker (Supp Fig. 2J); the N-terminal portion includes several arginine residues that are methylated to regulate SMAD6 activity [48], while the C-terminal portion contains the MH2 protein-interacting domain [49]. Since both major domains are required for the regulatory role of SMAD6 [50], we hypothesized that SMAD6 rescue of homeostatic endothelial cell alignment downstream of Notch required full-length SMAD6. Reduced Notch signaling, either via Notch1 or RPBJ depletion, led to endothelial cell misalignment in response to homeostatic flow that was not rescued by expression of constructs encoding only either the N-terminal or C-terminal portion of SMAD6 (Fig. 3c–f). These data indicate that full-length SMAD6 is required to mediate the effects of Notch signaling on homeostatic endothelial cell flow alignment.

### SMAD6 regulates endothelial cell proliferation

To better understand the effects of reduced SMAD6 function on flow-mediated endothelial cell responses, we examined the transcriptome of endothelial cells under homeostatic laminar flow relative to non-flow conditions, and with depleted Smad6 levels, via RNA-seq analysis. Pearson Correlation Analysis revealed good correlation between experimental replicates of each condition (Supp. Fig. 3A). Principle Component Analysis (PCA) distinguished transcriptomes of control (non-targeting siRNA) and SMAD6 depleted endothelial cells, and transcriptomes also clustered by flow status (Supp. Fig. 3B). Overall comparisons (33,694 genes) showed that, when binned by flow status, only 1.2% of transcripts were significantly up- or down-regulated with reduced Smad6 levels under non-flow (static) conditions, while 6.9% of transcripts changed with reduced Smad6 levels under homeostatic flow conditions (Supp. Table 1). When binned by depletion condition, control endothelial cells under static vs. flow conditions had 6.7% of transcripts showing expression level changes, while Smad6 KD cells had 8.9% of transcripts changed in static vs. flow conditions. These numbers suggest that the magnitude of flow-mediated changes is greatest in endothelial cells with reduced Smad6 levels, consistent with a role for SMAD6 in endothelial cell flow responses.

Despite the fact that flowed EC with Smad6 KD morphologically resembled non-flowed EC [NT (static) vs. Smad6 KD (flow)], 7.9% of analyzed genes (2655/33,694) differed in relative expression between these gene sets, even more than the 6.7% that differed between static and flow without Smad6 manipulation (Supp. Table 1). Finally, the greatest change in gene expression (11.2%) was seen when comparing flowed normal EC to non-flowed EC with Smad6 KD (Supp. Table 1). We next looked at individual gene sets, and found that a significant number of genes normally flow-responsive (up or down) became non-responsive with Smad6 KD (1041/2262, 46%), and that an even larger group of genes that were normally non-responsive to flow became flow-responsive (up or down) with Smad 6 KD (1782) (Supp. Fig. 3C–F shows top 50 genes/category; Supp Fig. 3G shows overlap). Finally, many genes that appeared concordant in direction of flow-responsiveness between control and Smad6 KD had baseline (non-flow) changes (up or down) that led to expression changes under flow in Smad6 KD relative to control (data not shown). We conclude that reduced SMAD6 levels have multiple impacts on the EC transcriptome under homeostatic laminar flow: (1) dampened expression changes for some flow-responsive genes; (2) about half of flow-responsive genes becoming non-responsive; and (3) aberrant up- and down-regulation of genes usually not responsive to flow. This analysis supports that SMAD6 is a key regulator of endothelial cell flow responses.

Smad6 expression is upregulated in lung EC of adult mice relative to infants [51], suggesting a role in vascular quiescence. Consistent with this idea, Gene Ontogeny (GO) Analysis to identify cellular processes significantly affected by Smad6 depletion revealed that transcripts associated with the cell cycle were enriched in endothelial cells with reduced Smad6 levels, independent of flow status (Supp Fig. 3H). We hypothesized that endothelial cell proliferation is negatively regulated by SMAD6, and we found that expression of the proliferation marker Ki67 was increased with Smad6 depletion under both static and homeostatic flow conditions (Supp Fig. 3I, J). BrdU incorporation, which labels S-phase cells, was increased upon Smad6 depletion in both static conditions and trending upward under flow conditions (Supp Fig. 3K, L). Thus, repression of endothelial cell proliferation that is a prerequisite of vascular quiescence requires SMAD6.

### SMAD6 regulates endothelial cell barrier function and junctions

**Fig. 4:**
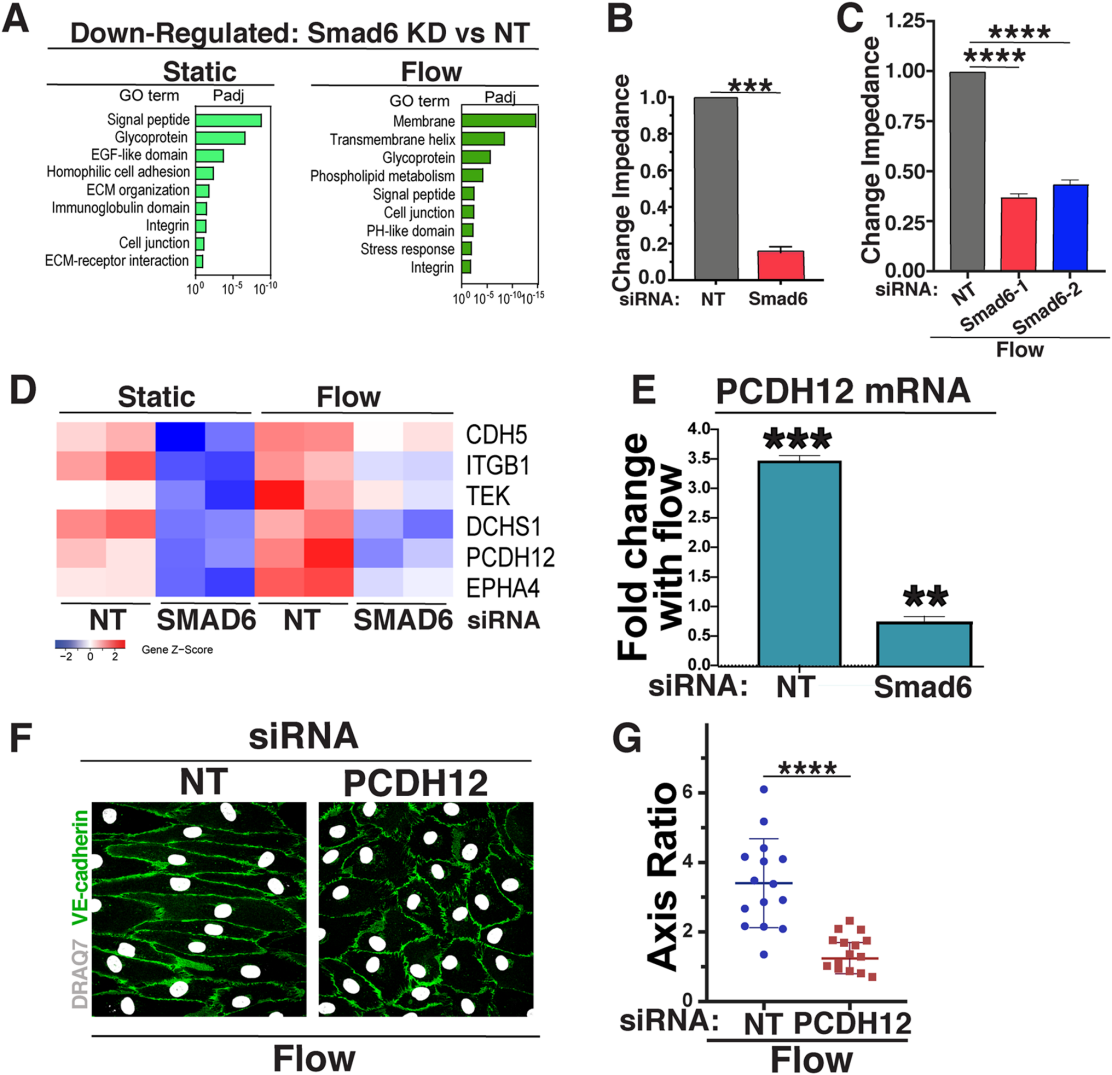
SMAD6 regulates endothelial cell barrier function and cell–cell junction genes under homeostatic flow. **a** Gene ontology (GO) analysis performed on differentially expressed genes from bulk RNA-seq data sets using DAVID. GO terms significantly enriched (*p* adjusted < 0.1) in down-regulated are shown. **b** Change in impedence after 24 h of EC with indicated siRNAs under static (control) conditions, normalized to control. Statistical analysis, One-way ANOVA; ***p ≤ 0.001. **c** Change in impedence after 72 h of EC with indicated siRNAs under flow, normalized to control. Statistical analysis, One-way ANOVA; ****p ≤ 0.0001. **d** Heat map showing subset of cell–cell-adhesion genes (see text for details) down-regulated with reduced Smad6 levels under indicated conditions. **e** qPCR of relative PCDH12 RNA levels under flow relative to static (control) with indicated siRNAs. **f** Representative panels of HUVEC stained with VE-cadherin (green, junctions) and DAPI (white, nucleus) under flow conditions with indicated treatments. White arrow, flow vector. Scale bar, 50 μM. **g** Quantification of cell axis ratio. Statistical analysis, student’s *t*-test; ****p ≤ 0.0001. *N* = 3, representative experiment shown

GO Analysis also indicated that expression of genes associated with cell–cell junctions was down-regulated in endothelial cells with reduced Smad6 levels (Fig. 4a). Since SMAD6 regulates junction morphology in the absence of flow [42], we hypothesized that the barrier formed by endothelial cell–cell junctions and important for proper vascular function was compromised by SMAD6 depletion. Endothelial cells with depleted Smad6 levels had reduced barrier function relative to controls, as measured by trans-endothelial electrical impedance (Fig. 4b), and the defective resistance downstream of reduced Smad6 levels was also significant under flow conditions (Fig. 4c).

To further examine how SMAD6 manipulations affect endothelial cell–cell junctions, we focused on expression differences in cell–cell adhesion and junction genes between control and Smad6 KD endothelial cells under both static and flow conditions. Overlapping genes from these two genelists were identified, and a small subset with significant overall expression levels was examined further. Most junction genes were upregulated with homeostatic laminar flow in controls, and relative expression of a subset of these genes was reduced in endothelial cells depleted for Smad6, regardless of flow status. The net effect was that a group of cell junction genes had reduced expression in Smad6-depleted endothelial cells relative to contols when both were exposed to homeostatic laminar flow (Fig. 4d, compare Flow-NT to Flow-Smad6 KD). Of these, the protocadherin PCDH12 was chosen for further analysis, since PCDH12 is selectively expressed in arterial endothelial cells, and its deletion leads to changes in murine arterial blood pressure, while human mutations are associated with brain arterial calcification [52–54]. Validation of PCDH12 expression changes via qRT-PCR showed significant upregulation of PCDH12 expression with homeostatic laminar flow, and this increase was blunted to 60% of static control levels in flowed endothelial cells with reduced Smad6 levels (Fig. 4e). We next asked whether PCDH12 functions in flow alignment responses, by subjecting HUVEC treated with PCDH12 siRNA to homeostatic laminar flow, and found that PCDH12 depleted endothelial cells failed to align (Fig. 4f, g; Supp Fig. 3M). These results show that SMAD6 is required for full PCDH12 expression in response to flow, and that PCDH12 expression is necessary for proper endothelial cell alignment under homeostatic flow conditions. These findings suggest that SMAD6 regulation of PCDH12 contributes to endothelial cell homeostatic flow-mediated responses downstream of Notch-induced mechanotransduction.

## Discussion

This work reveals a functional requirement for SMAD6 in endothelial cell responses to homeostatic laminar flow, which is the shear stress experienced by arterial endothelial cells and is considered atheroprotective. SMAD6 functions downstream of Notch1; since Notch1 is a mechanotransducer, it is likely that shear stress signals transduced by Notch1 signaling mediate flow responses that maintain vascular homeostasis in part through SMAD6. Expression profiling reveals numerous SMAD6-dependent changes in endothelial gene expression under flow, including downregulation of cell cycle/proliferation genes and upregulation of cell-cell adhesion genes, consistent with a role for SMAD6 in regulating the maintenance of endothelial cell quiescence and morphogenesis in response to laminar flow. Thus SMAD6 is a critical transducer of Notch-dependent endothelial cell flow responses that promote the maintenance of vascular homeostasis.

Notch regulation of homeostatic flow responses is linked to its regulation of SMAD6 expression levels. Loss of Notch1 signaling significantly reduced Smad6 RNA levels under homeostatic flow conditions, and restored expression of SMAD6 was sufficient to rescue flow-mediated alignment downstream of loss of Notch signaling. Interestingly, Notch-dependent endothelial cell alignment under homeostatic flow also utilizes the ligand Dll4 and requires Notch-mediated transcription (this study), while initial flow-induced endothelial cell responses require Notch but not its transcriptional activity [22], although subsequent flow responses may depend on Notch transcriptional activity [21]. These findings suggest that equilibration to laminar flow involves a switch from a non-transcriptional to a transcriptional program. Consistent with a the idea of a switch, analysis of endothelial cell junction changes under flow showed immediate effects on VE-cadherin clustering, followed by later junction and cell shape changes [11]. A switch model is also consistent with SMAD6 expression regulation being critical for vascular homeostasis in response to flow, and suggests that endothelial cells may have evolved different mechanisms for an acute response to changes in mechanotransduction vs. equilibration to ongoing mechanotransduction inputs.

SMAD6 functions as a negative regulator of BMP signaling, and canonical BMP signaling also affects flow responses in complex ways, so SMAD6 may regulate flow responses downstream of both Notch and BMP inputs. Alternatively, SMAD6 also has BMP-independent functions in innate immunity and other cellular processes [55–57], so SMAD6 may also affect endothelial cell flow responses in BMP-independent ways. Moreover, it is likely that other pathways involved in endothelial cell flow responses, such as KLF2 and/or KLF4, function upstream or downstream of Notch and SMAD6 [58]. In any case, expression profiling showed that SMAD6 manipulations change transcriptional endothelial cell flow responses in both directions, and more genes change expression when SMAD6 levels are depleted under homeostatic flow conditions (6.9%) vs. non-flow conditions (1.2%), indicating that homeostatic flow amplifies SMAD6-dependent transcriptional differences in endothelial cells. While some of the genes whose expression profiles change with Smad6 manipulations have Smad-binding motifs in their promoters, we hypothesize that most gene expression changes are downstream of initial Smad-binding and indirect, as the readout is homeostatic flow after 72 h of equilibration.

Loss of SMAD6 led to a more “activated” endothelial cell phenotype, with cell cycle/proliferation pathways upregulated and cell–cell junction pathways down-regulated, and these changes were accompanied by significant loss of barrier function. These findings are consistent with SMAD6 regulating the atheroprotective endothelial cell quiescence phenotype that accompanies flow-mediated alignment under homeostatic flow and important for barrier function, and it is also consistent with the hemorrhage phenotype of *Smad6*^*−/−*^ mutant embryos [42] and the increase in Smad6 expression in lungs of adult mice relative to infants [51]. Interestingly, cell cycle pathways were also upregulated in endothelial cells with reduced levels of Notch1 [21], consistent with SMAD6 being downstream of Notch activation. Although expression profiling suggests that endothelial cell–cell adhesion changes with reduced SMAD6 are likely to be complex and involve multiple adhesion receptors, the arterial-expressed protocadherin PCDH12 was significantly upregulated by homeostatic laminar flow, and this upregulation was dramatically blunted by Smad6 depletion. Since independent reduction of PCDH12 levels prevents endothelial cell flow-mediated alignment, it is likely that PCDH12 is one SMAD6 target normally upregulated downstream of SMAD6 under homeostatic flow that contributes to endothelial cell barrier function and quiescence. Thus, our data support a model whereby homeostatic flow-mediated mechanotransduction from Notch1 regulates expression of the effector SMAD6, and SMAD6 levels affect endothelial cell alignment, proliferation and barrier function to maintain vascular homeostasis. These findings provide new pathway intersections, and potential new therapeutic targets for diseases such as atherosclerosis that are linked to loss of vascular homeostasis.

## Methods

### Cell culture

HUVEC (Lonza, #C2519A) and HAEC (Lonza, #C2535) were maintained according to manufacturer’s recommendations and used at passage 2–4. For HUVEC and HAEC culture, EBM-2 (Endothelial Cell Growth Basal Medium-2, Lonza, #CC-3156) was supplemented with EGM-2 SingleQuots Supplements (Lonza, #CC-4176) (called EBM-2+). Main experiments were independently replicated with a different lot of HUVEC, and key experiments were replicated in HAEC at least two independent times. HUVEC and HAEC were certified mycoplasma-free by the UNC Tissue Culture Facility.

### Endothelial cell flow experiments

HUVEC and HAEC were plated at 100% confluency in each lane of a µ-Slide VI^0.4^ (Ibidi, #80601) coated with fibronectin (Millipore Sigma, F2006, 5μg/mL) 4 h prior to the experiment in EGM-2+ medium. After 2 h for cell attachment and spread, slides were washed 3× in flow medium [EBM-2 with 10% FBS (Gibco, #26140-079) and 1× Antibiotic-Antimycotic (Gibco, #15240-062)], and incubated in flow medium for 2 h prior to flow onset. Uniform laminar shear stress was generated by attaching slide chambers to a pump system (Ibidi, #10902) with both the slide and the pump apparatus kept at 37°C and 5% CO_2_. To reduce cell shearing, flow was applied for 30 min at 5 d/cm^2^, then 30 min at 10 d/cm^2^, followed by 72 h at 15 d/cm^2^. For siRNA knockdown, siRNA incubation was for 30 h prior to plating and flow initiation.

### Endothelial cell shear stress measurements

All data presented under “flow” conditions is at 15 d/cm^2^ laminar flow for 72 h (Ibidi system) and considered to be homeostatic laminar flow. At least 10 cells/condition were measured, and each cell is represented as an individual data point. Experiments were replicated at least three times.

#### Cell axis ratio

Cell shape and alignment were measured by staining for VE-cadherin or PECAM1 as described below. Each cell size measurements were recorded by measuring total cell length and width using ImageJ. Cell axis ratio was length divided by width, with length the direction of flow.

#### Nuclear displacement angle

Nuclear displacement angle was measured using the angle measurement tool in Image J. Measured angles were degrees separating a line perpendicular to the flow vector and a line through the nucleus along its long axis (see Supp. Fig. 1C).

#### Cell polarization

We determined the Golgi or centrosome location relative to the nucleus. HUVEC and HAEC stained for the Golgi (GM130) or the centrosome (TUBGCP2) and nuclei (DAPI) were analyzed by dividing the nucleus into equal thirds and and binning the organelle relative to the flow vector: upstream, middle, or downstream. At least five fields/experiment were quantified per condition.

### Endothelial cell transfection

HUVEC (Lonza) were transfected with non-targeting siRNA (NT, Life Technologies, #4390847) or experimental siRNAs [single siRNAs or siRNA pools—Supp. Table 2 (Key Resources)] using the standard Lipofectamine 2000 (Invitrogen, 11668027) manufacturer's protocol. Briefly, for each siRNA to be tested: 24 μl of 10uM siRNA was diluted in 476 μl of opti-MEM media and separately, 24 μl of Lipofectamine 2000 reagent was diluted in 476 μl of opti-MEM medium. Each mixture was incubated separately 5 min at RT, then mixed 15 min at RT. This mixture was added to a 10cm plate of ~70% confluent HUVEC or HAEC in EGM-2+ medium without antibiotics (EBM-2 media, added BulletKit without gentamicin supplement) and incubated 24 h at 37 °C, then incubated for 6 h in fresh non-antibiotic EGM-2+ prior to plating in flow channels to start the experiment. Smad6-tdTomato was transfected into HUVEC as described [40] and provided approximately three to eightfold increase in baseline expression levels.

### Immunofluorescence

HUVEC and HAEC were fixed for 10 min in 4% PFA, washed 3× with PBS, then permeabilized for 10 min in 0.1% Triton X-100. Cells were blocked for 30 min at RT in 1% BSA (Millipore Sigma, #A-4503) in PBS, then incubated with primary antibodies (1:100—see Supp. Table 2) in 1% BSA for 45 min at RT. After washing 5× with 1% BSA, samples were incubated with Alexa-fluor-conjugated anti-species secondary antibodies (1:250—see Supp. Table 2) plus Alexa-fluor-conjugated phalloidin (1:50—Invitrogen, #A12379 or #A12381) and DAPI or DRAQ7 (1:300—Sigma #10236276001 or Abcam #ab109202, respectively) in 1% BSA for 30 min at RT. Samples were washed 5× in 1% BSA and mounted by washing 3× in 80% glycerol (Millipore Sigma, #G5516) in PBS. Immunofluorescent imaging was done either immediately after mounting or slides were wrapped in foil at 4°C for up to 2 weeks.

BrdU incorporation was an adaptation [59]. Briefly, HUVEC were incubated with 10 μM BrdU (Millipore Sigma, #B5002) in flow media for the last 90 min of flow and fixed in ice-cold 100% methanol. Samples were blocked for 30 min at RT in 1% BSA, then acid treated as follows: 10 min in cold (4°C) 1N HCl on ice, rinse in 2N HCl and 10 min at RT, then incubated in 10mM citric acid solution (pH 7.4, in 0.2M Na^+2^HPO_4_) for 10 min at RT, rinsed with 1% BSA 3× and processed for antibody staining as detailed above with a sheep polyclonal antibody to BrdU (1:100—Abcam, #ab1893), then Alexa Fluor Donkey anti-sheep 594 secondary (1:250—Life Technologies #A-11016), along with DAPI and phalloidin as described above.

All fluorescent imaging was done using an Olympus FV3000 Laser Scanning Confocal Microscope and Flow View software. Olympus OIB file formats were imported into ImageJ using Bio-Formats Importer20 for analysis and quantification.

### Quantitative RT-PCR

Primers are listed in Supp. Table 2. cDNA was generated from 1 μg mRNA using iScript reverse transcription kit (Bio-Rad, #1708891) and diluted 1:3 in water. qRT–PCR was performed using iTaq Universal SYBR Green SuperMix (Bio-Rad, #1725121). SYBR Green real-time PCR was performed in triplicate on the Applied Biosystems QuantStudio 6 Flex Real-Time PCR System. For quantification, relative expression of each gene to GAPDH in each sample was calculated by 2^(CT of gene−CT of GAPDH). Statistical significance was determined by one-sample *T*-test compared to a reference value of onefold change.

### RNA sequencing and analysis

RNA was extracted using TRIzol (Invitrogen) from two biological replicates (independent experiments), and TruSeq Stranded mRNA Library Prep Kit (Illumina) was used to prepare cDNA and Illumina libraries for sequencing (HiSeq4000). 2–5 × 10^7^ 50-bp paired-end reads per sample were obtained and mapped to human genome GRCh38-1.2.0 downloaded from https://support.10xgenomics.com/single-cell-gene-expression/software/pipelines/latest/advanced/references with TopHat/2.1.1 using default settings. Mapping rate was >92% for all samples, and gene expression was determined with Htseq-count/0.6.1 using the union mode (https://htseq.readthedocs.io/en/master/). PCA analysis was performed with the top 400 genes selected by largest weight (loading) contribution to PCs 1, 2 or 3 using the R package SINGuLAR. Differential expression analysis was performed with DESeq2 in R, and lists of differentially expressed genes were obtained (FDR < 0.05). Heat maps were generated using the heatmap.2 function in the ‘gplots’ package in R. Gene ontology analysis was performed using the DAVID functional annotation tool version 6.8 (https://david.ncifcrf.gov/). All gene ontology terms shown in this study have a corrected P value (the “Benjamini” value from DAVID) < 0.1.

### Barrier function analysis

#### Real-time cell analysis (RTCA) experiments

Barrier properties were measured using a commercially available system [xCELLigence Real-Time Cell Analyzer (RTCA)]; Acea Biosciences/Roche Applied Science, Basel, Switzerland). RTCA measures electrical impedance as a readout for the barrier status of cells grown on top of microelectrode coated surfaces. HUVEC were pre-treated with siRNAs or drug for 24 h prior to plating an equal cell number onto the microelectrode surface of the E-plate (E-plate 16, Roche Applied Science). Impedance readings were taken automatically every 5 min for 24 h.

#### Electric cell-substrate impedance sensing system (ECIS) experiments

Endothelial barrier function analysis under static and flow conditions was performed using impedance-based cell monitoring using ECIS zeta theta (Applied Biophysics) in conjunction with the Ibidi pump system. HUVEC were seeded onto an ibidi flow chamber with 8 microelectrodes on the bottom (ECIS Flow Array 1E). Experiments proceeded exactly as ibidi flow experimental setup, with the addition of impedance readings taken every five min across each electrode for 72 h.

### Statistical analysis

All statistical analyses were performed using Prism v8.01 (www.graphpad.com), with an *α* of 0.05. For two-sample data sets with equal variances (control -v- a single experimental condition) unpaired, two-tailed Student’s *t*-test was used as reported in figure legends. For data sets with greater than two conditions and equal variances, one-way analysis of variance (ANOVA) with Tukey’s *post-hoc* test was used as reported in figure legends. *p ≤ 0.05, **p ≤ 0.01, ***p ≤ 0.001, ****p ≤  0.0001. *ns* not significant.

## Supplementary Information

Below is the link to the electronic supplementary material.

Supplementary file1 (PDF 3471 kb)

## Data Availability

The RNA-seq data that support the findings of this study are available in the Gene Expression Omnibus (GEO) under the accession number GSE147036.
