# Supplementary material for: SMAD6 transduces endothelial cell flow responses required for blood vessel homeostasis
Source: Angiogenesis. 2021 Mar 29;24(2):387–98. doi: 10.1007/s10456-021-09777-7 (PMC8206051; doi:10.1007/s10456-021-09777-7)
Supplement: Supplementary file 1 — Supplementary file1 (PDF 3471 kb) [file 10456_2021_9777_MOESM1_ESM.pdf]

SUPPLEMENTARY FIGURES AND FIGURE LEGENDS

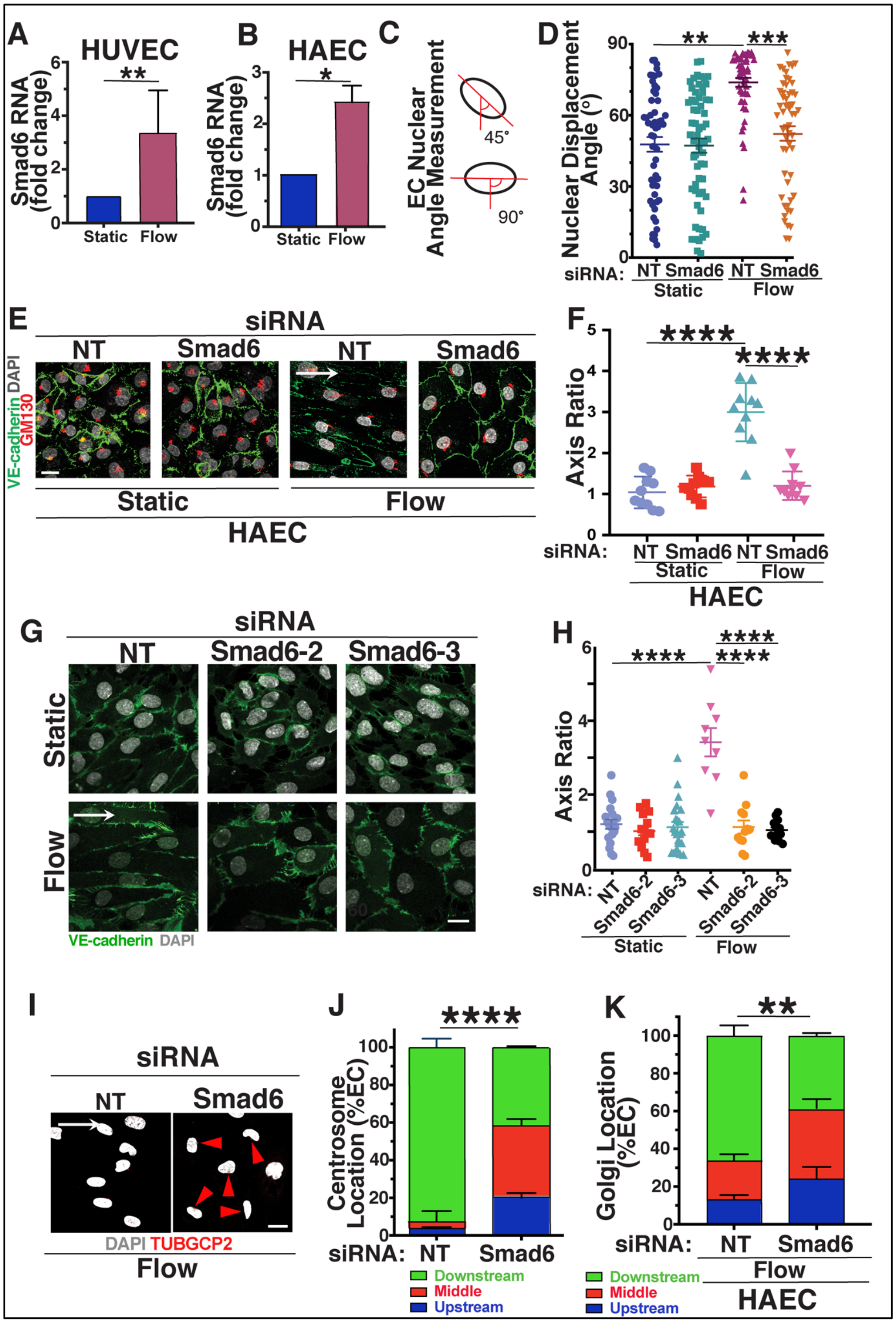

Supplemental Figure 1 (Linked to Fig 1). SMAD6 is Required for Homeostatic Endothelial Cell Flow-Mediated Alignment and Polarization.

Supplemental Figure 1 (Linked to Fig 1). SMAD6 is Required for Homeostatic Endothelial Cell Flow-Mediated Alignment and Polarization.

A,B) Smad6 qPCR RNA levels (normalized to static control) in HUVEC (A) and HAEC (B). Statistical analysis, Student's t-test; *, p≤0.05; **, p≤0.01. **C)** Diagram of nuclear angle measurements used to quantify flow alignment. **D)** Nuclear angle quantification of **Fig1A**. N=3, representative experiment shown. **E)** Representative panels of HAEC stained with VE-cadherin (green, junctions), GM130 (red, Golgi), and DAPI (white, nucleus) under control (static) or flow conditions with indicated treatments. White arrow, flow vector. Scale bar, 20μM. **F)** Quantification of cell axis ratio of HAEC in indicated conditions from **Supp Fig 1E**. Statistical analysis, One-way ANOVA; ****, p≤0.0001. N=3, representative experiment shown. **G)** Representative panels of HUVEC with additional SMAD6 siRNAs (see **Supp. Table 2**) stained with VE-cadherin (green, junctions) and DAPI (white, nucleus) under control (static) or flow conditions. White arrow, flow vector. Scale bar, 20μM. **H)** Quantification of cell axis ratio in indicated conditions from **Supp Fig 1G**. Statistical analysis, One-way ANOVA; ****, p≤0.0001. N=3, representative experiment shown. **I)** Representative panels of HUVEC stained with TUBGCP2 (red, centrosome) and DAPI (white, nucleus) under flow conditions with indicated treatments. White arrow, flow vector. Red arrowhead, centrosome location. Scale bar, 20μM. **J**) Quantification of centrosome location in **Supp Fig 1I** relative to nucleus in indicated conditions. n = ≥30 cells per condition. Statistical analysis, One-way ANOVA; ****, p≤0.0001. **K)** Quantification of HAEC Golgi localization relative to nucleus (**Supp Fig 1E**) in indicated conditions. n = ≥30 cells per condition. Statistical analysis, One-way ANOVA; **, p≤0.01.

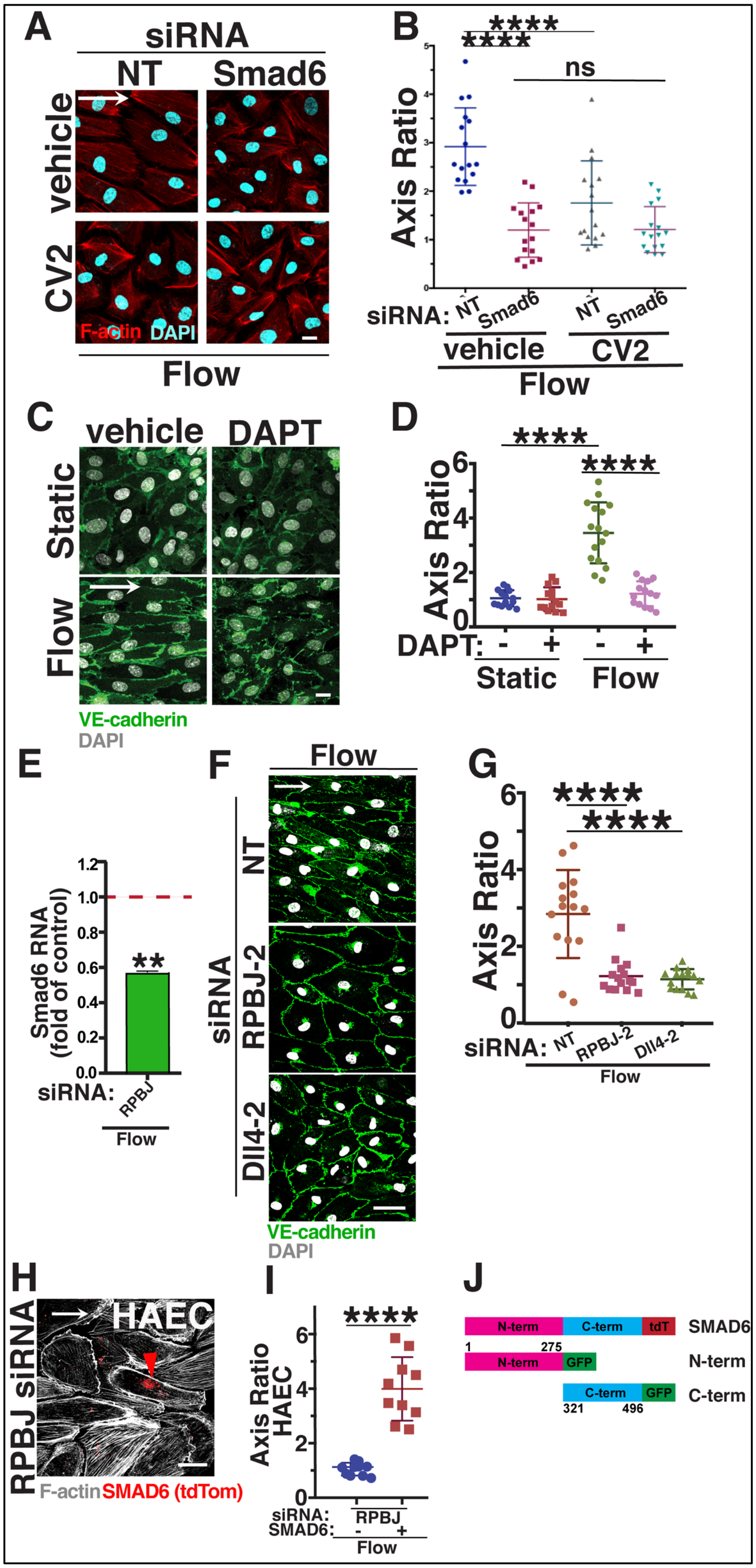

Supplemental Figure 2 (Linked to Fig 1, Fig 2, Fig 3). SMAD6 is Downstream of Notch Signaling in Homeostatic Endothelial Cell Flow-Mediated Alignment.

Supplemental Figure 2 (Linked to Fig 1, Fig 2, Fig 3). SMAD6 is Downstream of Notch Signaling in Homeostatic Endothelial Cell Flow-Mediated Alignment.

A) Representative panels of HUVEC stained with Phalloidin (red, actin) and DAPI (white, nucleus) under flow conditions with indicated treatments. White arrow, flow vector. Scale bar, 20μM. **B)** Quantification of cell axis ratio in indicated conditions. Statistical analysis, One-way ANOVA; ****, p≤0.0001; ns, not significant. N=3, representative experiment shown. **C)** Representative panels of HUVEC stained with VE-cadherin (green, junctions) and DAPI (white, nucleus) under control (static) or flow conditions with indicated treatments. White arrow, flow vector. Scale bar, 20μM. **D)** Quantification of cell axis ratio in indicated conditions. Statistical analysis, One-way ANOVA; ****, p≤0.0001. N=3, representative experiment shown. **E)** qPCR RNA levels (normalized to static control) in HUVEC treated with RPBJ siRNA. Statistical analysis, Student's t-test; **, p≤0.01 **F)** Representative panels of HUVEC with additional RBPJ and DLL4 siRNAs (see **Supp. Table 2**) stained with VE-cadherin (green, junctions) and DAPI (white, nucleus) under control (static) or flow conditions. White arrow, flow vector. Scale bar, 50μM. **G)** Quantification of cell axis ratio in indicated conditions. Statistical analysis, One-way ANOVA; ****, p≤0.0001. N=3, representative experiment shown. **H)** Representative panel of HAEC treated with RPBJ siRNA and stained with phalloidin (F-actin, white) and expression construct (SMAD6) under flow conditions. White arrow, flow vector. Red arrowhead, positive EC. Scale bar, 20μM. **I)** Quantification of cell axis ratio in indicated conditions. Statistical analysis, student's t-test; ****, p≤0.0001. N=3, representative experiment shown. **J)** Diagram showing SMAD6 constructs. Numbers indicate amino acids in human SMAD6.

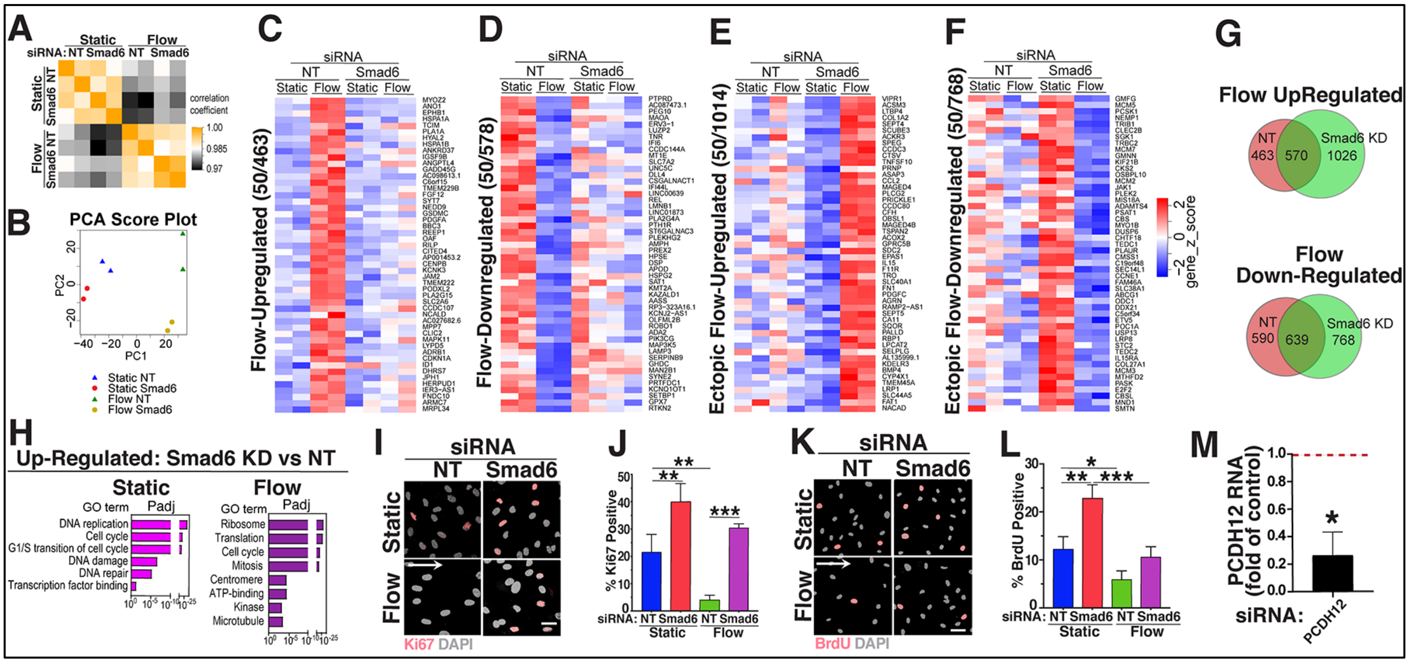

Supplemental Figure 3 (Linked to Fig 4). Endothelial Cell Transcriptomics Reveals Increased Proliferation with Reduced SMAD6 Levels.

A) Pearson Correlation Analysis of bulk RNA-seq data from endothelial cells of indicated conditions. Correlation coefficients between any two samples plotted as heatmap. **B**) Principle Component (PC) Analysis of bulk RNA-seq data. **C-F)** Heatmaps showing relative expression levels of genes (top 50 by p-value, totals in labels) whose flow pattern changed with Smad6 KD: **C)** genes up-regulated with flow, unresponsive with Smad6 KD; **D)** genes down-regulated with flow, unresponsive with Smad6 KD; **E)** genes unresponsive in NT but up-regulated with flow and Smad6 KD; **F)** genes unresponsive in NT but down-regulated with flow and Smad6 KD. NT, non-targeting siRNA. **G)** Venn diagrams showing overlap of genes up- and down-regulated under flow in controls vs. Smad6 KD. **H**) GO analysis performed on differentially expressed genes from bulk RNA-seq data using DAVID. Representative GO terms significantly enriched (P adjusted < 0.1) in up-regulated genes are shown. **I)** Representative panels of HUVEC stained for Ki67 (pink, proliferation marker) and DAPI (white, nucleus) under control (static) or flow conditions with indicated siRNA treatments. White arrow, flow vector. Scale bar, 20uM. **J)** Quantification of Ki67+ cells in indicated conditions. Statistical analysis, One-way ANOVA; **, p≤0.01; ***, p≤0.001. N=3, representative experiment shown. **K**) Representative panels of HUVEC labeled with BrdU and stained with anti-BrdU (pink, S-phase marker) and DAPI (white, nucleus) under control (static) or flow conditions with indicated siRNA treatments. White arrow, flow vector. Scale bar, 20μM. **L)** Quantification of BrdU incorporation in indicated conditions. Statistical analysis, One-way ANOVA; *, p≤0.05; **, p≤0.01; ***, p≤0.001. N=3, representative experiment shown. **M)** qPCR RNA levels (normalized to NT control) in HUVEC treated with PCDH12 siRNA. Statistical analysis, Student's t-test; *, p≤0.05.

Supplemental Table 1. Changes in HUVEC Gene Expression (total 33,694).

COMPARISON: Endothelial Cell (Condition)	Genes UP Number (%)	Genes DOWN Number (%)	Total Genes DEG No (%)
NT (static) vs. Smad6 KD (static)	164 (0.5%)	240 (0.7%)	404 (1.2%)
NT (static) vs. NT (flow)	1033 (3.1%)	1229 (3.6%)	2262 (6.7%)
NT (flow) vs. Smad6 KD (flow)	1361 (4.0%)	958 (2.9%)	2319 (6.9%)
Smad6 KD (static) vs. Smad 6 KD (flow)	1596 (4.7%)	1407 (4.2%)	3003 (8.9%)
NT (static) vs. Smad6 KD (flow)	1286 (3.8%)	1369 (4.1%)	2655 (7.9%)
NT (flow) vs. Smad6 KD (static)	1850 (5.5%)	1903 (5.7%)	3753 (11.2%)

Supplemental Table 2. Key Resources.

Reagent	Source	Catalog
Antibodies:		
Rabbit monoclonal anti-VE-cadherin	Cell Signaling	D87F2;2077969
Mouse monoclonal anti-PECAM1	Cell Signaling	3528S;2160882
Rabbit monoclonal anti-GM130	Abcam	EP892Y;52649
Mouse monoclonal anti-TUBGCP2	Abcam	GCP2-01;140225
Mouse monoclonal anti-γ tubulin	Thermo Fisher	T5326;2211251
Sheep polyclonal anti-BRDU	Abcam	ab1893;302659
Rabbit polyclonal anti-Ki67	Abcam	ab15580;443209
Goat anti-Rabbit 488	Life Tech	A-11034
Goat anti-Mouse 584	Life Tech	A-11005
Donkey anti-Sheep 594	Life Tech	A-11016
Critical Commerical Assays:		
Lipofectamine 2000 Transfection	Thermo Fisher	11668027
Lipofectamine LTX Transfection	Thermo Fisher	15338030
HUVEC Nucleofector Kit	Lonza	VPB-1002
Amaxa Nucleofector Kit Primary Mam	Lonza	VPI-1001
iScript Reverse Transcription Kit	Bio-Rad	1708891
iTaq Universal SYBR Green SuperMix	Bio-Rad	1725121
μ-Slide VI0.4	Ibidi	80601
xCELLigence RTCA E-Plate 16	Acea Biosci	5469830001
qPCRprimers(5'-3'):		
fGAPDH:CAGCAAGAGCACAAGAGGAAGAGA	Eurofins	N/A
rGAPDH:TTGATGGTACATGACAAGGTGCGG	Eurofins	N/A
fSMAD6:CTGGAGTTGTTGAGCAGCC	Eurofins	N/A
rSMAD6:GTGCGTCTTTCTTGTTTTGTCC	Eurofins	N/A
fKLF4:CCCAATTACCCATCCTTCCT	Eurofins	N/A
rKLF4:CTTTGGCTTGGGCTCCTCT	Eurofins	N/A
fNOTCH1:GTCAACGCCGTAGATGACC	Eurofins	N/A
rNOTCH1:TTGTTAGCCCCGTTCTTCAG	Eurofins	N/A
fRBPJ:GGATAGGAAATAGTGACCAAGAAATG	Eurofins	N/A
rRBPJ:AGTGCTTTCGCTTGTCTGAG	Eurofins	N/A
fDLL4:TGCAACTGCCCTTATGGCTTTGTG	Eurofins	N/A

rDLL4:ACAAGTTGTTCATGGCTTCCCT GC	Eurofins	N/A
fJAG1:TGCCAAGTGCCAGGAAGT	Eurofins	N/A
rJAG1:GCCCCATCTGGTATCACACT	Eurofins	N/A
fJAG2:TGGGACTGGGACAACGATA	Eurofins	N/A
rJAG2:ATGCGACACTCGCTCGAT	Eurofins	N/A
fHES1:ACGTGCGAGGGCGTTAATAC	Eurofins	N/A
rHES1:GGGGTAGGTCATGGCATTGA	Eurofins	N/A
fPCDH12:CAAGCATCCACGTCACATG G	Eurofins	N/A
rPCDH12:GTGTGGCATTGGTTAGCAA CA	Eurofins	N/A
Single siRNAs:		
SMAD6-1	Life Tech	s8411
SMAD6-2	Life Tech	s8410
NOTCH1-1	Life Tech	9635
RBPJ-1	Life Tech	s7252
DLL4-1	Life Tech	s29214
JAG1-1	Life Tech	s1175
JAG2-1	Life Tech	s7643
PCDH12-1	Life Tech	s27870
siRNA Pools:		
SMAD6-3	Santa Cruz	sc-38380
NOTCH1-2	Santa Cruz	sc-36095
RBPJ-2	Santa Cruz	sc-38214
DLL4-2	Santa Cruz	sc-39667
JAG1-2	Santa Cruz	sc-37202
JAG2-2	Santa Cruz	sc-39672
PCDH12-2	Santa Cruz	sc-76896
